# Health outcomes and drug utilisation in children with Noonan syndrome: a European cohort study

**DOI:** 10.1186/s13023-025-03594-7

**Published:** 2025-02-17

**Authors:** Michele Santoro, Ingeborg Barisic, Alessio Coi, Joachim Tan, Ester Garne, Maria Loane, Ljubica Odak, Maria Valentina Abate, Elisa Ballardini, Clara Cavero-Carbonell, Miriam Gatt, Mika Gissler, Kari Klungsøyr, Nathalie Lelong, David Tucker, Diana Wellesley, Joan K. Morris

**Affiliations:** 1 Unit of Epidemiology of Rare Diseases and Congenital Anomalies, Institute of Clinical Physiology, National Research Council, Via Moruzzi 1, 56124 Pisa, Italy; 2 Children’s Hospital Zagreb, Centre of Excellence for Reproductive and Regenerative Medicine, Medical School University of Zagreb, Zagreb, Croatia; 3 NIHR Great Ormond Street Hospital Biomedical Research Centre, UCL GOS Institute of Child Health, London, UK; 4 School of Health and Medical Sciences, City St George’s University of London, London, UK; 5 Department of Paediatrics and Adolescent Medicine, Lillebaelt Hospital, University Hospital of Southern Denmark, Kolding, Denmark; 6 Faculty of Life and Health Sciences, Ulster University, Northern Ireland, UK; 7 Department of Medical and Laboratory Genetics, Endocrinology and Diabetology with Daily Care Unit, Children’s Hospital Zagreb, Zagreb, Croatia; 8 Neonatal Intensive Care Unit, University Hospital of Ferrara IMER Registry (Emilia Romagna Registry of Birth Defects, Department of Medical Sciences, University of Ferrara, Ferrara, Italy; 9 Rare Diseases Research Unit, Foundation for the Promotion of Health and Biomedical Research in the Valencian Region, Valencia, Spain; 10 Directorate for Health Information and Research, G’Mangia, Pietà, Malta; 11 Department of Knowledge Brokers, THL Finnish Institute for Health and Welfare, Helsinki, Finland; 12 Region Stockholm, Academic Primary Health Care Centre, Stockholm, Sweden; 13 Department of Molecular Medicine and Surgery, Karolinska Institutet, Stockholm, Sweden; 14 Department of Global Public Health and Primary Care, University of Bergen, Bergen, Norway; 15 Division of Mental and Physical Health, Norwegian Institute of Public Health, Bergen, Norway; 16 Université de Paris, CRESS-Epopé, INSERM, INRA, Paris, France; 17 Public Health Wales, Swansea, UK; 18 Wessex Clinical Genetics Service, University Hospital Southampton, Southampton, SO16 5YA UK

**Keywords:** Noonan syndrome, Cohort, Survival, Hospitalization, Surgeries, Prescriptions

## Abstract

**Background:**

Noonan Syndrome (NS) is a rare multisystemic disorder with heterogeneous phenotypic manifestations. The aim of this study was to analyse rates of survival, hospitalisation, surgeries and prescriptions in children born with NS in the first 10 years of life.

**Methods:**

This is a multi-centre population-based cohort study. Data on 175 liveborn children diagnosed with NS from 11 EUROCAT congenital anomaly registries were linked to healthcare databases. Each registry applied a common data model to standardise data and run common syntax scripts to produce aggregated results which were pooled using random effects meta-analyses.

**Results:**

Mortality rates were high in the first year of life with 5.4% (95%CI 1.5%-10.1%) of children dying before the age of 1 year with a further 2% dying up to age 5. In the first year, 87.9% (95%CI 75.3%-94.3%) of children were hospitalized and the median Length Of hospital Stay (LOS) was 15.3 days (95%CI 9.3–21.2). After the first year, the proportion of children hospitalized remained higher than 70%, but the LOS decreased to 1.3 days per year. In the first 5 years, 65.2% of children underwent a median of two surgical procedures. The median age at first surgery was 29 weeks. The proportion of children with an antibiotic prescription increased from 53.6% at age 1 to 62.4% yearly until 4 years of age.

**Conclusions:**

Children with NS have high mortality and morbidity not only in the first year of life but also up to five years of age. This study evaluated the health burden of NS and provided information for clinicians, health-care providers and families.

## Background

Noonan Syndrome (NS) (MIM # 605,275) is typically an autosomal dominant multisystem disorder with heterogeneous phenotypic manifestations which represent a major burden for affected patients and their families [1]. NS belongs to the family of RASopathies, a group of conditions caused by gain-of-function pathogenic variants in genes encoding components or regulators of the RAS/mitogen-activated protein kinase signalling pathway [2, 3]. Clinical characteristics may include distinctive facial features, broad or webbed neck, congenital heart defects, chest deformity, renal anomalies, cryptorchidism, lymphatic malformations, bleeding diathesis, short stature and developmental delay/learning difficulties. The phenotype is variable and mildly affected individuals may not be diagnosed until adulthood. Diagnosis is typically based on clinical features, but molecular genetic testing can confirm the diagnosis in about 80% of cases [4–10]. It is necessary that different paediatric subspecialties are familiar with the health burden and course of NS to ensure appropriate parental counselling, multidisciplinary monitoring and treatment.

Children and adults with NS need to receive regular medical care and follow-up to monitor and manage their health [6, 7, 10]. Medications may be prescribed, but specific information on prescriptions issued to individuals with NS is lacking. [12–15]

The mortality rate can vary depending on several factors, including the severity of cardiac abnormalities that are present in 50 to 80% of individuals [9, 16]. According to a study that followed a large cohort of individuals with RASopathies, the overall mortality is relatively low [17]. However, certain cardiac complications, such as Hypertrophic CardioMyopathy (HCM), can increase the risk of mortality [17, 18]. A report on patients with NS and HCM showed a significantly worse risk-adjusted late survival rate compared to individuals with nonsyndromic HCM [19]. Additionally, a study analysing outcomes in children with NS and HCM found that patients with NS and HCM had a worse risk profile at presentation, resulting in significant early mortality [20]. Additional population-based studies are needed to further understand the mortality risk and long-term outcomes in population-based cohorts of newborns with NS.

Hospitalisation may be necessary for those who experience complications related to their condition, such as congenital heart defects, feeding problems and failure to thrive in the first year of life. Some individuals with NS may require surgical procedures [16, 21]. It is suggested that NS is associated with increased hospital stays for paediatric cardiac surgery which has implications for costs. One study found that NS accounted for 1.6% of paediatric cardiac surgery admissions, and patients with NS tended to have longer hospital stays, higher total charges, and increased inpatient mortality compared to those without NS [22]. However, there is still little evidence to provide specific information based on a population-based approach about hospitalisation and surgery rates in general for all individuals with NS with or without congenital heart defects.

The study presented here is part of the EUROlinkCAT project that aimed to investigate the health outcomes of European children born with major Congenital Anomalies (CA) by linking data from population-based registries to electronic health care databases [23].

The aim of this study was to analyse rates of survival, hospitalisation, surgeries and prescriptions in children born with NS in the first 10 years of life.

## Methods

This is a population-based data-linkage cohort study. Data were available on children born with NS (ICD10 BPA code Q87.14 and ICD9 BPA code 759.896) in the period 1995–2014 from 11 European Surveillance of Congenital Anomalies (EUROCAT) registries [24, 25] in 7 European countries. EUROCAT registries collect standardised data on all major CA cases diagnosed by the first year of age in their population using multiple sources of ascertainment according to EUROCAT guidelines [26].

Data from participating registries were linked to data in local healthcare databases covering the registry's geographical area (mortality, hospital admission and discharge data, prescription data). Linked data on outcomes of interest were included up to the child’s 10th birthday or to 31st December 2015 (whichever was earlier). A detailed description of the linkage methods used in the EUROlinkCAT project has been published elsewhere [23, 27, 28]. Only years with good quality healthcare data and a high proportion of successful data linkage were included in the study period and the overall successful linkage was higher than 95% [27].

For the investigation of survival, data on mortality were obtained through electronic linkage with vital statistics and mortality databases used in the regions and countries covered by the registries. A detailed description of the methods has been published elsewhere. [29–31]

For the analysis of hospitalisation and surgical procedures, cohort data were linked to the hospital databases used in the geographical areas covered by the participating registries. Reference populations were all liveborn children without CAs from the same population covered by the registry, in the same birth years. The registries in Tuscany and Northern Netherlands used a random sample of their population (10% and 20%, respectively) matched by date of birth and sex. No reference children were available for the three English registries. For the registries of Paris, Norway and Malta, data on hospitalization and surgical procedures were not available for this study. Rates of hospitalisation, excluding hospital admissions associated with birth only, for children with NS were compared with those in the reference population and also to children born with any major CA previously estimated by the EUROlinkCAT project [32].

Data on prescribed/dispensed medications was available by linking cohorts to local electronic prescription databases from the year 2000. Data on selected medications (antibiotics, asthma, cardiac, anti-seizure and insulin) were included in the study. Prescription data were available for 5 registries (Finland, Emilia Romagna, Tuscany, Valencian Region and Wales). Data from Wales included prescriptions for medications issued by a General Practitioner (GP), whereas data from the other four regions included medications prescribed by clinicians/GP and dispensed by a pharmacy. All registries used Anatomical Therapeutical Chemical (ATC) coding except for Wales which used Read coding which was converted to ATC coding using a look-up table in SAIL (Secure Anonymised Information Linkage Databank). No registry had information on hospital inpatient prescribing.

### Statistical analysis

Each registry used a common procedure for data collection, standardisation, quality control and statistical analyses, as defined in the EUROlinkCAT project [23]. Data from each registry were analysed locally by the registry using common Stata syntax scripts. Aggregated data and analytic results were uploaded to a secure Central Results Repository based at Ulster University, UK and then transferred to the research team using a secure web platform.

Kaplan–Meier survival analyses were performed by each registry to account for censoring i.e. children who were lost to follow-up due to death or emigration from the study area or who had not reached their 10th birthday by 31st December 2015. The Kaplan–Meier survival estimates with 95% confidence intervals (95%CI) from each registry were then combined in a random-effects meta-analysis using a modified method by Combescure et al. [33] to obtain pooled estimates. Survival estimates were calculated at the following ages: 1 week, 4 weeks and 1, 5 and 10 years.

Similarly, each registry used a Kaplan–Meier analysis to estimate the following indicators of hospitalisation: percentage of children (i) hospitalised; (ii) hospitalised with a hospital stay longer than 10 days for term-born children; (iii) undergoing surgery. The overall estimates by age group were calculated using a random effects meta-analysis.

The median Length Of Stay in hospital (LOS) per year, defined as the number of days spent in hospital between the date of admission and the date of discharge, was calculated within each registry and random effects meta-analyses were performed using the “metamedian” package in R (version 4.0.3) for the following age groups: < 1 year, 1–4 years, 5–9 years. The same method was used to obtain pooled estimates of the median age at first surgery and the median number of surgical procedures for the following age groups: < 1 year, 1–4 years, and 0–4 years, as not all registries had data on children up to the child’s 10th birthday.

Finally, the proportion of children receiving a prescription per year and the median number of prescriptions per year were calculated by age groups.

## Results

Data on a total of 175 children born with NS between 1995 and 2014 came from 11 EUROCAT registries listed in Table 1.

**Table 1:** Contributing European Surveillance of Congenital Anomalies (EUROCAT) registries, included birth years

Participating registries	Included birth years
Finland^e^	1995–2014^b^
France: Paris^a,d^	1995–2014
Italy: Emilia Romagna	2008–2014
Italy: Tuscany	2005–2014
Malta^a,d,e^	1995–2014
Norway^a,d,e^	1999–2014
Spain: Valencian Region	2007–2014^c^
UK: East Midlands and South Yorkshire^d^	2003–2012
UK: Thames Valley^d^	2005–2013
UK: Wales^e^	1998–2014
UK: Wessex^d^	2004–2014

^a^data on hospitalisation and surgical procedures not available

^b^study period for hospitalisation and surgical procedures 1997–2014

^c^study period for hospitalisation and surgical procedures 2010–2014

^d^data on prescriptions not available

^e^whole nation covered

### Survival

A total of 15 deaths were observed in the cohort during the study period (Table 2). Twelve deaths occurred in the first year of life with an infant mortality rate of 5.4% (95% CI 1.5%–10.1%) and an additional 3 deaths were observed up to age 5. Survival estimates ranged from 98.0% (95% CI 95.9%–100.0%) at 1 week to 92.4% (95% CI 87.0%–98.1%) at 10 years.

**Table 2:** Pooled survival estimates at selected age groups up to 10 years of age for children born with Noonan syndrome (*n* = 175), 1995–2014

Age	N. of deaths	Survival %	95% CI
1 week	4	98.0	95.9–100.0
4 weeks	6	96.7	93.9–99.6
1 year	12	94.6	90.9–98.5
5 years	15	92.6	87.4–98.1
10 years	15	92.4	87.0–98.1

#### Hospitalisations

**Fig. 1:**
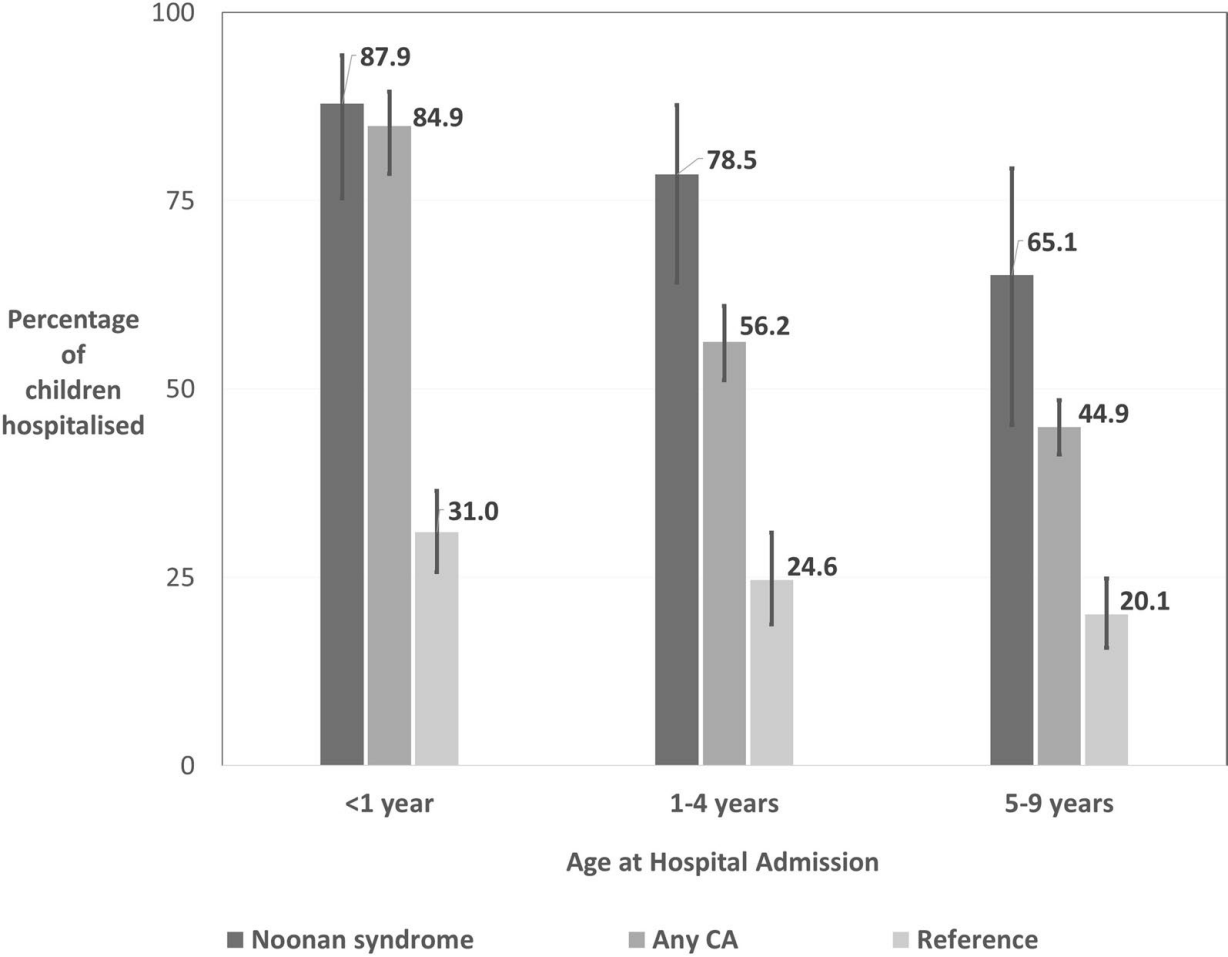
Percentage hospitalised (with 95% CIs) children with Noonan syndrome, children with any congenital anomaly (‘Any CA’) and children without a congenital anomaly (‘Reference’), by age, 1995–2014

Overall, 87.9% (95% CI 75.3%–94.3%) had at least one hospital stay in the first year of life (Table 3), which decreased to 65.1% per year (95% CI 45.2%–79.3%) at age 5–9 years. The percentage of children with NS who were hospitalised was three times higher than the reference population at all ages. After the first year of life, the proportion is also higher than that of children with any major CA (Fig. 1). In the first year of life, 31.4% (95% CI 17.2%–46.8%) of term-born children had at least one single hospital stay longer than 10 days. The proportion decreased after the first year.

**Table 3:** Percentage hospitalised, percentage hospitalised with a long stay (≥ 10 days) and median length of stay per year of children with Noonan syndrome (*n* = 145), by age, 1995–2014

Age (year)	Children with any hospitalisation			Children with a length of stay over 10 days^a^			Median length of stay	
	n	%	95% CI	n	%	95% CI	days	95% CI
< 1	126	87.9	75.3–94.3	35	31.4	17.2–46.8	15.3	9.3–21.2
1–4	104	78.5	64.1–87.7	17	15.8	3.2–37.0	1.3	0.3–2.2
5–9	52	65.1	45.2–79.3	3	–	–	0.5	0.0–1.0

^**a**^Only children born ≥ 37 weeks of gestation were included

The median LOS was 15.3 days (95% CI 9.3–21.2) in children with less than 1 year of age and dramatically decreased after the first year of age.

### Surgery and other procedures

Overall, 65.2% (95% CI 41.0%−81.5%) of children underwent surgery in the first 5 years of life and 34.8% in the first year (Table 4). The median number of surgical procedures in the first 5 years was 2 (95% CI 1.3–2.7). The median age at first surgery was 29.0 weeks (95% CI 13.6–84.4).

**Table 4:** Proportion of children with Noonan syndrome (*n* = 145) undergoing surgery and median number of surgeries by age, 1995–2014

Age (year)	N children undergoing surgery	% children undergoing surgery	95% CI	Median number of surgeries	95% CI
< 1	50	34.8	21.1–49.0	1.3	0.7–1.8
1–4	60	50.8	22.3–73.7	2.0	1.7–2.3
0–4	80	65.2	41.0–81.5	2.0	1.3–2.7

### Prescriptions

About 50% of children had at least one anti-asthmatic prescription during the first 10 years of life. The percentage was quite stable across the age group (Table 5). Cardiac medications were prescribed to about 22% of children during the study period. The percentage of children with cardiac medication in the first year was 10.7% and it was 2.9% yearly in children aged 5–9. Antibiotics were prescribed to 53.6% of the children in the first year and the proportion increased with increasing age. After the first year of age, in fact, 62.4% of children had on average two prescriptions every year until 4 years of age. After four years of age, the percentage of prescriptions decreased. In the investigated cohort, only one child had an anti-seizure medication.

**Table 5:** Percentage of children with a prescription and median number of prescriptions by age and type of prescription, 2000–2014

Medication	Age (year)	Percentage of children with a prescription in a year	95% CI	Median number of prescriptions each year	IQR
Any asthma medication (ATC code R03)	< 1	17.9	11.3–26.2	2	2–2
	1–4	24.7	20.5–29.4	2	1–4
	5–9	18.2	13.8–23.2	3.5	2–5
Any cardiac medication (ATC code C01-C03, C07-C09, excluding C01BA51, C01BA71, C01CA24)	< 1	10.7	50.7–18.0	8.5	5.5–9.5
	1–4	8.4	50.8–11.7	6	4–10
	5–9	2.9	10.2–5.5	4.5	4–5
Antibacterials for systemic use (ATC code J01-J05)	< 1	53.6	43.9–63.0	2	1–4
	1–4	62.4	57.3–67.3	2	1–4
	5–9	38.4	32.7–44.4	1.5	1–3

IQR = Interquartile range

## Discussion

In our study, we estimated a survival rate of 92.4% at 10 years which is somewhat lower than the estimate calculated in a study on patients affected by RASopathies by Calcagni et al. [17] This can be explained by the fact that our cohort consisted of children with NS diagnosed in infancy according to EUROCAT guidelines, which represented more severe cases of NS. The survival in NS is dependent on the severity of the phenotype, particularly the severity of the heart defect. This study shows that the prognosis significantly improves after one year of age.

The percentage of hospitalized children with NS was high in all age groups, especially in the early years of life. Factors that may contribute to longer hospital stays in the first years of life for patients with NS include feeding difficulties, respiratory tract infections, and the presence of congenital heart defects, [16, 34–36]. Cardiac abnormalities are present in most patients with NS and may require cardiac interventions, leading to extended hospital stays. Feeding problems and failure to thrive in the first year of life are very common in infants with NS. Another EUROlinkCAT study estimated that about 8% of NS patients need gastrostomy for tube feeding indicating that feeding problems may be a major cause of long hospital stays in infancy [37].

A study found that paediatric cardiac surgery admissions tended to be 4.5 days longer and cost $54,296 more in total charges for patients with NS compared to those without the syndrome. Inpatient mortality was also increased [22]. Additionally, patients with NS are more likely to experience complications such as chylothorax, or perioperative bleeding from coagulation defects which can further prolong hospitalization. The increased complexity of surgical procedures and the need for concomitant procedures and re-interventions, also cause longer hospital stays [22, 38–40]. This helps to explain our findings that the percentage of hospitalised children with NS was higher compared to other children with or without CA in all age groups. Assessment of the total hospital care needs is important for a proper and efficient planning of healthcare services.

Individuals with NS may require surgery to address specific medical conditions or complications associated with the syndrome. Different types of surgery that may be performed in individuals with NS include cardiac surgery, airway surgery, orthognathic surgery, orchidopexy and neurosurgical interventions. Cardiac surgery may be necessary to repair or replace heart valves or correct other structural abnormalities [16, 38] Some individuals with NS may have airway abnormalities, such as lymphangiomatosis of the pleura, lungs, and chest wall, which can lead to complications like chylothorax. Airway surgery may be performed to address these issues [41]. Corrective jaw surgery may be performed in individuals with NS who have craniofacial abnormalities. This surgery can help improve facial symmetry and correct malocclusion [42]. In rare cases, individuals with NS may require neurosurgical interventions for conditions such as Chiari malformation, syringomyelia, craniosynostosis or brain tumours [43]. The results of our study show surgical procedures are often performed and that two-thirds of children with NS have undergone more than one surgical procedure in the first 5 years of life. The expected frequency of surgery is an important information for healthcare providers, clinicians and parents to children with NS.

Regarding the results of the selected categories of medications investigated in the EUROlinkCAT project [23], we found that the prevalence of prescription is quite high for antibiotics in early infancy. In particular, the use of antibiotics in children with NS increased after the first year of life, whereas the rates of prescription of antibiotics in the general population estimated in some European countries decreased after the first year [44, 45]. This may indicate more frequent infectious diseases in children with NS at that age.

Due to the chronic nature and severity of congenital heart defects, the median number of prescriptions per year was the highest for cardiac medications. The decreasing prevalence of prescriptions of cardiac medications is expected as most children with NS have less severe congenital heart defects, such as ventricular septal defects or pulmonary valve stenosis, which rarely need any medical treatment after the first year.

### Strengths

The main strength of this study was the population-based design which included all children diagnosed in the first year of life with NS in the areas covered by the participating EUROCAT registries and not only children referred to tertiary care centres. The population-based setting allows the calculation of more representative estimates of health outcomes. Additionally, we used data collected and validated by EUROCAT registries which use standardised definitions and coding of major CAs. Pooling data from 11 registries in 8 European countries allows powerful investigation of a rare syndrome like NS. Finally, health outcomes were estimated using an innovative methodology based on standardized procedures of analysis and the use of a meta-analytic approach. [23, 46]

### Limitations

A limitation of the study was that failing to link to the records in the healthcare databases could have produced bias in the estimates of some health outcomes. However, the overall successful linkage was higher than 95% and similar to that observed for the reference population (i.e. liveborn children without any congenital anomaly). Furthermore, linkage failure is most likely to occur in the first days of life before the newborns have their permanent name or Identification number as showed in a previous EUROlinkCAT study [27]. As survival in the first day is high in children born with NS, this limitation is likely to have a minor impact. Another limitation of the study is that we were not able to analyse data separately by type of associated anomalies due to the very small sample size in most of the participating registries. Also, we were not able to analyse the diagnosis for the hospital stays. However, the results provide information on what parents to children with NS can expect in relation to mortality and morbidity up to 10 years of age. Finally, data on hospital inpatient prescriptions were not available for the investigated groups of medications and this might lead to a slight underestimation.

## Conclusions

This multicentre population-based study reported on rates of survival, hospitalisations, and prescriptions of children born with NS in eleven areas of seven European countries. Survival at 1 and 10 years was 95% and 92%, respectively. In the first year of life one-third of children had a long hospital stay. After the first year, hospitalisation was still high, but the median length of stay dramatically decreased. Two-thirds of children had a surgical procedure in the first five years of life with a medium of two interventions. The results of the study provide information to evaluate the health burden for children diagnosed with NS that is important for parental counselling and the planning of healthcare services. Efforts must be made to support families throughout the childhood of children born with NS.

## Data Availability

The data that support the findings of this study are available from the participating registries of congenital anomalies, but restrictions apply to the availability of these data, which were used under license for the current study, and so are not publicly available. Data are however available from the authors for scientifically valid requests and with permission of the participating registries of congenital anomalies.

## Abbreviations

NS: Noonan syndrome
HCM: Hypertrophic cardiomyopathy
CA: Congenital anomalies
ICD-10 BPA: International Statistical Classification of Diseases, Tenth Revision, with British Paediatric Association 1- digit extension
ICD-9 BPA: International Statistical Classification of Diseases, Ninth Revision, with British Paediatric Association 1- digit extension
LOS: Length of stay in hospital
ATC: Anatomical Therapeutical Chemical
SAIL: Secure Anonymised Information Linkage
